# Repeated Labilization-Reconsolidation Processes Strengthen Declarative Memory in Humans

**DOI:** 10.1371/journal.pone.0023305

**Published:** 2011-08-05

**Authors:** Cecilia Forcato, María L. C. Rodríguez, María E. Pedreira

**Affiliations:** Laboratorio de Neurobiología de la Memoria, Departamento de Fisiología, Biología Molecular y Celular, Facultad de Ciencias Exactas y Naturales, Universidad de Buenos Aires, IFIBYNE – CONICET, Buenos Aires, Argentina; Harvard Medical School, United States of America

## Abstract

The idea that memories are immutable after consolidation has been challenged. Several reports have shown that after the presentation of a specific reminder, reactivated old memories become labile and again susceptible to amnesic agents. Such vulnerability diminishes with the progress of time and implies a re-stabilization phase, usually referred to as reconsolidation. To date, the main findings describe the mechanisms associated with the labilization-reconsolidation process, but little is known about its functionality from a biological standpoint. Indeed, two functions have been proposed. One suggests that destabilization of the original memory after the reminder allows the integration of new information into the background of the original memory (memory updating), and the other suggests that the labilization-reconsolidation process strengthens the original memory (memory strengthening). We have previously reported the reconsolidation of human declarative memories, demonstrating memory updating in the framework of reconsolidation. Here we deal with the strengthening function attributed to the reconsolidation process. We triggered labilization-reconsolidation processes successively by repeated presentations of the proper reminder. Participants learned an association between five cue-syllables and their respective response-syllables. Twenty-four hours later, the paired-associate verbal memory was labilized by exposing the subjects to one, two or four reminders. The List-memory was evaluated on Day 3 showing that the memory was improved when at least a second reminder was presented in the time window of the first labilization-reconsolidation process prompted by the earlier reminder. However, the improvement effect was revealed on Day 3, only when at least two reminders were presented on Day2 and not as a consequence of only retrieval. Therefore, we propose central concepts for the reconsolidation process, emphasizing its biological role and the parametrical constrains for this function to be operative.

## Introduction

It is generally recognized by cognitive psychology [1], [2] that memory can be rebuilt at retrieval. In this field, a profound analysis of human memories clearly suggests that they are not constant through time. Indeed, they vary not only in content but also in strength (i.e. flashbulbs memories, [3]; misleading post-event information [4]). The presentation of some components associated with the acquisition of the original memory triggers its retrieval. Consequently, it is possible that the current recollection of facts or new information could be interconnected and modified by the retrieval of the original memory.

On the other hand, in the neurobiological field understanding of learning and memory is quite different. Thus, the process of transforming new information into long-lasting memory was the object of interest in neurobiology throughout the last century. The seminar studies of Muller and Pilzecker [5] using verbal learning led to the idea that memories become enduring through a process of consolidation. This theory assumes that memories are labile during a time window after acquisition but, as time passes, memories become stable and resistant to amnesic agents. The consolidation process has been described using behavioral, pharmacological and molecular approaches in diverse species from nematodes to humans. The general outcome assumes that consolidation is a conserved evolutionary process that requires an initial phase of RNA and protein synthesis [6]–[12]. However, the idea that memories are immutable after consolidation has been challenged. Since the early study of Misanin et al. [13] several reports have shown that after the presentation of a specific reminder, reactivated old memories become labile and again susceptible to amnesic agents. Such vulnerability diminishes with the progress of time and implies a re-stabilization phase, usually referred to as reconsolidation. It has been proposed that reconsolidation shares many of the cellular and molecular mechanisms used during consolidation. From the extensive studies developed in the last decade, a general conclusion emerged. In fact, the term reconsolidation is not used to represent an exact recapitulation of initial consolidation, but rather the functional role of the process, which is to make memory stable again [14].

Considering the contributions of the different studies as a whole, the main findings describe the mechanisms associated with the labilization-reconsolidation process, but little is known about its functionality from a biological standpoint. In this context, what is the function of memory reconsolidation? Two non-mutually exclusive hypotheses have been proposed to address the question [15]. One states that destabilization of the original memory after the reminder allows the integration of new information into the background of the original memory and is referred to as memory updating [16], [17]. The other suggests that the labilization-reconsolidation process strengthens the original memory [18]. Taking into account the biological role of the phenomenon, we have proposed that reconsolidation is not triggered whenever a memory is retrieved, as a consequence, the identification of general and boundary conditions for reconsolidation are central topics. Thus, the strength of the memory trace, the age of the memory, the duration of the reactivation, which can shift from reconsolidation to extinction, and the discrepancy between expected and current events -mismatch- are parameters that determine the occurrence, or not, of reconsolidation [19]–[22]. Taking all these factors into consideration, a central conclusion is that the reconsolidation process is not triggered every time a memory is retrieved. Moreover, these boundary conditions determine the scenario for the analysis of the functionality of reconsolidation.

Interestingly, the hypothesis of updating has received experimental support using different paradigms and models. Morris et al. [23] looked into the idea that reconsolidation occurs in spatial memory when animals retrieve memory under circumstances in which new memory encoding is likely to occur. Therefore, they compared the effect of intrahippocampal administration of anisomycin in two contrasting conditions with respect to the presence or absence of new information. In situations where the state of the environment may be changed all the time, memory encoding remained engaged at the time of retrieval. So, the consolidated memory rendered labile and sensitive to intrahippocampal anisomycin impairing the re-stabilization of the trace. In line with these results and by the use of a reference memory task, Rodriguez et al. [24] showed that when the administration of anisomycin occurred before performance reached asymptote, the memory was labilized and its restabilization affected by the inhibition of the protein synthesis.

By a procedure that separates the learning of pure context from footshock-motivated contextual fear learning, Lee [25] demonstrated doubly dissociable hippocampal mechanisms of initial context learning and subsequent updating of the neutral contextual representation to incorporate the footshock. Thus, contextual memory consolidation was dependent upon BDNF expression in the dorsal hippocampus, whereas the footshock modification of the contextual representation required the expression of Zif268. In a Previous study [26] it was shown that these mechanisms were selectively involved in hippocampal memory consolidation and reconsolidation, respectively. Further, he demonstrated that memory reactivation is a necessary condition to modify memory content.

List-learning procedure has been used to assess reconsolidation in human episodic memory, Hupbach et al. [27] instructed subjects to memorize a list of objects and on a subsequent day they were primed to recall the learning episode (reactivation) before memorizing a second list. The authors interpreted that the reconsolidation effect was expressed as a significant number of intrusions from the second list to the target list.

But quite surprisingly, only two research papers have dealt with the strength function of reconsolidation. In one of them, Lee [26] found that a second learning trial strengthened a consolidated contextual fear memory, but only following its destabilization. Moreover, as we mentioned earlier, he demonstrated a double dissociation between cellular mechanisms of initial memory consolidation and reconsolidation. The first trial depended on BDNF, and the second, which strengthened memory through additional learning, depended on Zif-268. Interestingly, preventing memory destabilization invariably maintained the strength of the original memory. In the other, using a rat inhibitory avoidance, Inda et al. [28] tested whether reconsolidation mediates memory strengthening and its interaction with the passage of time. They found that successive reactivations of young memories, by re-exposition to the context, resulted in reconsolidation that mediated memory strengthening, an effect that was temporally limited.

We reported reconsolidation of a human declarative memory [29]. Our paradigm included five pairs of non-sense syllables, a cue syllable associated with a response syllable so that it would fit well as a semantic memory. However, the syllables were presented in a specific context consisting of light projected on a large screen, an image on the monitor screen and music. All these stimuli were shown keeping a temporal and spatial relation, that is, the paradigm included a temporo-spatial structure which defines a temporo-spatial context [30]–[31]. Based on the last description, the paradigm could be addressed as the combination of both semantic and episodic components of memory. In this paradigm the target memory was a list of five pairs of nonsense-syllables (L1), and the interfering agent was another list of syllables (L2). The main finding of this study was the demonstration that previously consolidated declarative memory returned to a labile state and became subject to stabilization again. This process of labilization-reconsolidation was triggered by the presentation of a cue-reminder (which included the context cues and one cue syllable, without giving the subjects the opportunity to write down the response syllable) which rendered the target memory labile again and created the possibility that a second training impaired the re-stabilization of the declarative memory within a defined time window (between 6 and 10 hours). In a second research paper [32] we evaluated whether our paradigm fulfils the two requirements that characterize the reconsolidation process, established previously in our invertebrate model [21], namely: the labilization of the reactivated memory and the specificity of the reminder structure. A series of experiments were performed with protocols similar to those used in the first study. Subjects were trained on two consecutive days on which they learned L1 and L2, respectively. A group of subjects received the cue-reminder before the L2-training on Day 2, while the other group only received the L2-training. Thus, it was confirmed that only the group that received the cue-reminder, which in turn labilized the memory, but not the other group which only went through the L2-training, showed significant deficits in L1-memory at testing on Day 3. On the other hand, we demonstrated that the impairment of L1-memory is no longer detected when the retrieval condition of the reminder was not accomplished. In one case, the change implied the presentation of the context cues alone (context-reminder) and, in the other, we excluded the mismatching component. Thus, in this last manipulation, the subjects had the possibility of writing down the response syllable (cue-response-reminder) and, consequently, the discordance was eliminated and the mismatch disappeared.

Finally, we began the analysis of the functionality of the reconsolidation process studying the incorporation of new information into a previously consolidated memory [33]. We demonstrated updating in the framework of declarative memory reconsolidation in humans. The updating occurs when the original memory is labilized by the presentation of the cue-reminder, and the verbal-instruction to incorporate the new information is given and the new information is shown. Under these conditions, the subjects were able to introduce this new information into the recalled declarative memory. Even more interestingly, although memory is labilized, the omission of the explicit order to add new information in the verbal instruction hinders the memory updating.

Here we deal with the strengthening function attributed to the reconsolidation process. To achieve this objective we triggered labilization-reconsolidation processes successively by repeated presentations of the cue-reminder. Participants learned an association between five cue-syllables (List) and their respective response-syllables. Twenty-four hours later, the paired-associate verbal memory was labilized by exposing the subjects to one, two or four cue-reminders. The List-memory was evaluated on Day 3 showing that the memory was improved when at least a second reminder was presented in the time window of the first labilization- reconsolidation process prompted by the earlier reminder. However, the improvement effect was revealed on Day 3, only when at least two cue- reminders were presented on Day2, and not as a consequence of only retrieval induced by the presentation of context cues or when the reminder did not include the mismatch. So, we propose central concepts for the reconsolidation process, emphasizing its biological role and the parametrical constrains for this function to be operative.

## Results

### Memory strengthening by repeated triggering of labilization- reconsolidation

**Figure 1:**
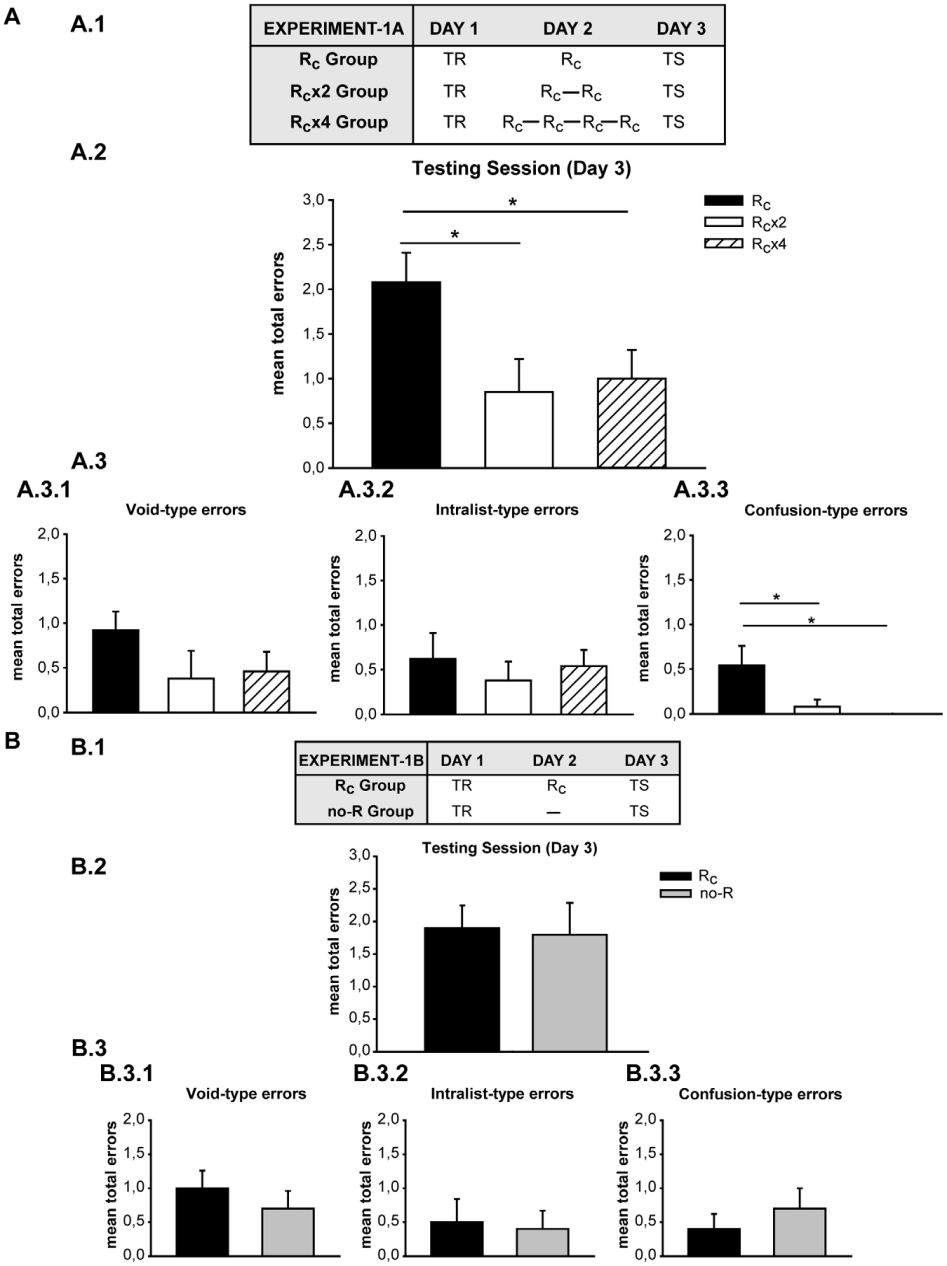
Memory strengthening by repeated triggering of labilization-reconsolidation. **A**) **Experiment 1A (n = 13). A.1) Experimental protocol.** A three-day experiment. TR, stands for the training session, R_c_ for the cue reminder, and TS for the testing session. Groups differ in the number of reminders that they received on Day 2. Group R_c_ received a cue reminder, Group R_c_x2 received two cue reminders, and Group R_c_x4 received the cue reminder four times. **A.2) Testing session.** Mean number of total errors +/- SEM on Day 3. *, p<0,05. Black bar stands for Group R_c_, white bar for Group R_c_x2 and stripe bar for the Group R_c_x4. **A.3) Error Type.** **A.3.1)** Mean number of Void-type errors +/− SEM on Day 3. **A.3.2)** Intralist-type errors **A.3.3)** Confusion-type errors. Symbols as above. **B**) **Experiment 1B (n = 10). B.1) Experimental protocol.** A three-day experiment. Symbols as in experiment 1A. Group R_c_ received a cue reminder on Day 2 and Group received no reminder. **B.2) Testing session.** Mean number of total errors +/− SEM on Day 3. Black bar stands for Group R_c_, grey bar for Group no-R. **B.3) Error Type.** **B.3.1)** Mean number of Void-type errors +/− SEM on Day 3. **B.3.2)** Intralist-type errors **B.3.3)** Confusion-type errors. Symbols as above.

In order to evaluate the possibility of memory strengthening by repeated reactivations, we did a three-day experiment with three groups (Figure 1A.1). On Day 1, subjects learned a list of five pairs of cue-response syllables (training session). On Day 2, they received a treatment session. The cue-reminder group received one cue-reminder (R_c_), the two cue-reminder group (R_c_x2) received two cue-reminders separated by a 5-min interval and the four cue-reminder group (R_c_x4) received the cue-reminder four times. The cue-reminder was formed by the specific context associated with the list plus one cue-syllable without the opportunity for subjects to write down the response syllable. This type of reminder triggers the labilization-reconsolidation process [27]. Finally, all subjects received the testing session on Day 3.

Moreover, we categorized the error-types made at testing. This categorization of the error-types allows us to distinguish the real effect of the strengthening on memory. That is, it is possible to define different scenarios for the enhancement and more importantly define how the precision of the memory was improved. First, a lower number of Void-errors would reflect that the volunteers could write down the correct response to a cue syllable which had previously not been answered. Second, a lower number of intralist errors could indicate an improvement in the accuracy of the association between the cue and response syllables in the List. Finally, the diminution in confusion-errors could also indicate strengthening in the precision of the memory, for example, allowing subjects to write down the three letters in the correct order.

#### Two or more cue-reminders improve performance on Day 3

ANOVA of repeated measures revealed no differences between the groups at training (Figure S1A, F(2,36) = 0,452, p = 0,640) as well as no group trial interaction (F(16,288) = 0,885, p = 0,587). Moreover, the analysis of the percentage of correct responses for the last four training trials disclosed no significant difference between the groups at the training session, (Figure S1A *inset*, F(2,36) = 1,043, p = 0,361).

The performance on Day 3 of each group was estimated by the mean of total errors made when responding to the cue-syllables of the two testing trials. Subjects that received two or four cue-reminders successively on Day2 performed better than those that received only one (Figure 1A.2). Specifically, the R_c_x2 and R_c_x4 groups made fewer errors than the R_c_ Group at the two testing trials (One-Way ANOVA F(2,36) = 3,854 p = 0,030; LSD post-hoc Comparison p = 0,015, p = 0,032 respectively).

It is necessary to stress that the hypothesis postulated here implies that repeated reactivations strengthen the memory, reflected as we have shown by a decrease in the number of total errors made at testing. Thus, to perform a more detailed analysis, the errors made at testing were classified in three categories. The first category as Void-types, when no response was written; the second as intralist-types when the response-syllable was not the right one but it belonged to the list; finally, as confusion-types when the response syllable was not included in the list. It is worthwhile noting the difference disclosed for the type of errors committed by each group (Figure 1A.3). Void type errors (blank responses, Figure 1A.3.1) were similar for the three groups (F(2,36) = 1,358 p = 0,270) like the intralist type (write down a response syllable for another cue syllable. Figure 1A.3.2) (F(2,36) = 0,255 p = 0,777). However, R_c_x2 and R_c_x4 groups made fewer confusion type errors (write down a nonexistent response syllable) than the R_c_ group. Indeed significant differences were revealed between groups at the two test trials (Figure 1A.3.3, One Way ANOVA F(2,36) = 4,868 p = 0,013; LSD post-hoc comparison p = 0,018, p = 0,007 respectively). This first result strongly suggests that the successively triggered labilization-reconsolidation improved the retention of a well acquired and consolidated declarative memory.

The standard method to reveal the role of reconsolidation implies that the disclosed effect of the treatment depends on memory reactivation [15], [34]. Thus, the comparison between a reactivated and a non-reactivated group is necessary. Accordingly, previous results showed that the presentation of one cue-reminder, during the treatment session, did not affect the performance at testing [29], [32]. In order to confirm this outcome, an additional experiment was carried out. The experiment included two groups: a cue reminder group (R_c_ group), which received a protocol similar to the one previously used, and a no reminder group (no-R Group). In this no-R Group, the training and testing sessions were comparable with the other group, but it did not receive a treatment session (Figure 1B.1). An ANOVA of repeated measures revealed no significant differences at training session between groups, F(1,18) = 0,000 p = 1,000; and no group per trial interaction, F(8144) = 0,678 p = 0,710 (Figure S1B). Moreover, the analysis of the percentage of correct responses for the last four training trials disclosed no significant difference between the groups at the training session, (Figure S1B *inset*, F(1,18) = 0,009, p = 0,926).

There were no significant differences between groups at testing (One Way ANOVA Figure 1B.2 F(1,18) = 0,039 p = 0,845). In addition, the comparison between the error types exhibited the same number of errors for each type in both groups (Figure 1B.3; Void-Type: F(1,18) = 0,669 p = 0,424; Intralist-Type: F(1,18) = 0,053 p = 0,820; Confusion-Type: F(1,18) = 0,622 p = 0,431).

Taken together, these results support the view that the repetition of the reminders and not their mere presentation improve the performance at testing.

### Successive retrievals do not strengthen the declarative memory

**Figure 2:**
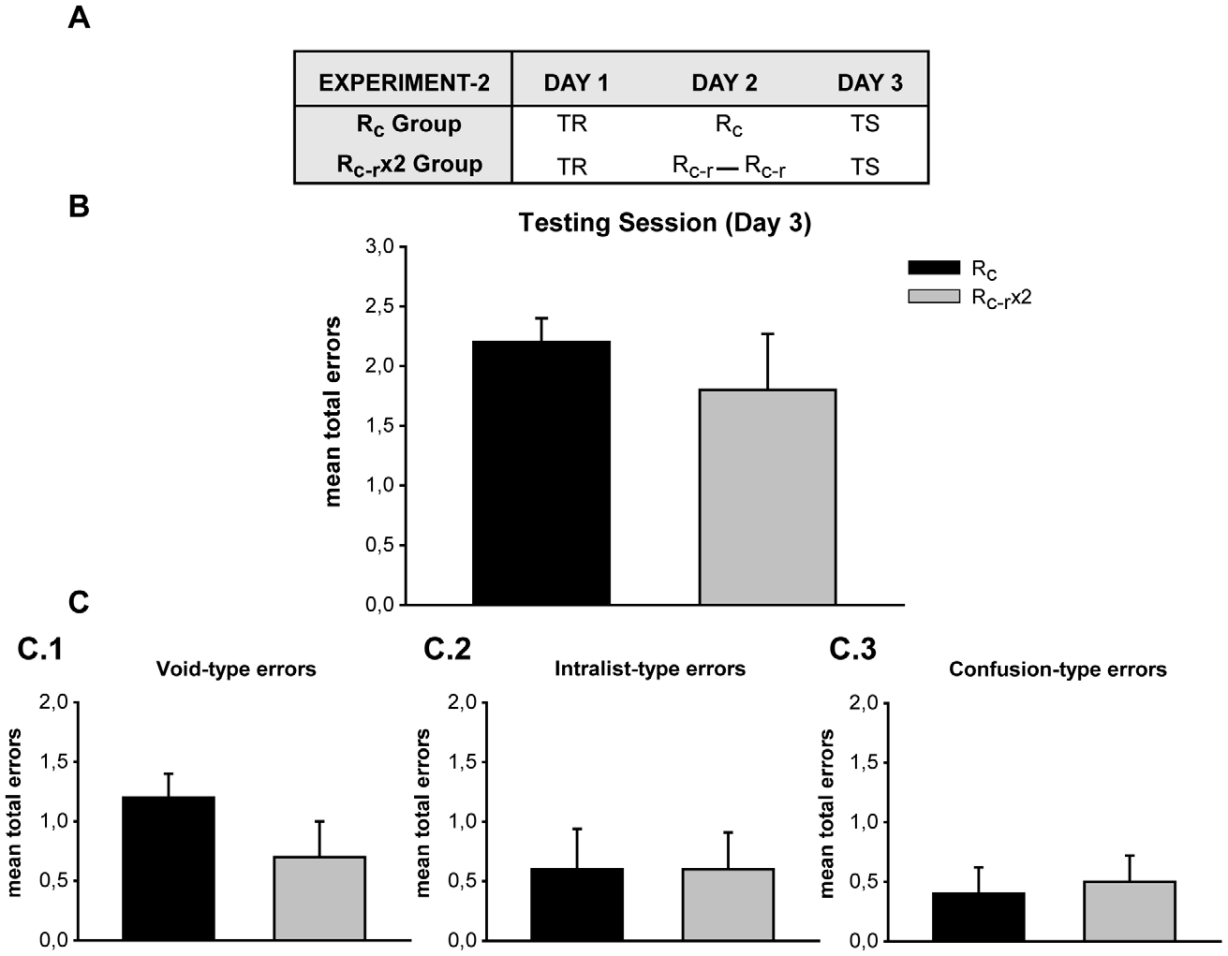
Experiment 2 (n = 10). Successive retrievals do not strengthen the declarative memory. **A**) **Experimental protocol.** A three-day experiment. Symbols as in experiment 1, R_c-r_ stands for the cue-response reminder. Group R_c_ received a cue reminder, Group R_c-r_x2 received the cue-response reminder twice. **B**) **Testing session.** Mean number of total errors +/− SEM on Day 3. Black bar stands for Group R_c_, grey bar for Group R_c-r_x2. **C**) **Error Type.** **C.1)** Mean number of Void-type errors +/− SEM on Day 3. **C.2)** Intralist-type errors **C.3)** Confusion-type errors. Symbols as above.

The results obtained above could be due to the effect of repeated retrievals instead of repeated destabilization of the original memory. To discard such an interpretation our model offers different reminders [28] to distinguish these contrasting interpretations, namely, that memory retrieval rather than memory reactivation reinforces the original memory. Indeed, we have shown that the omission of one of its parametrical conditions — such as the mismatching component in reminder cue-response group, R_c-r_ – retrieves memory but deactivates the reminder and, as a consequence, prevents the labilization of the target memory. Thus, to evaluate the effect of two successive retrievals on the strengthening of the target memory we performed a three-day experiment with two groups (Figure 2A). On Day 1, subjects learned the list of syllable-pairs (List). On Day 2, they received a treatment session. The cue-reminder group (R_c_) was exposed to one cue-reminder; the two cue-response reminders group (R_c-r_x2) received the cue reminders twice, separated by a 5-minute interval. Finally, all the subjects were tested on Day 3.

#### Two cue response reminders do not enhance performance on Day 3

**Figure 3:**
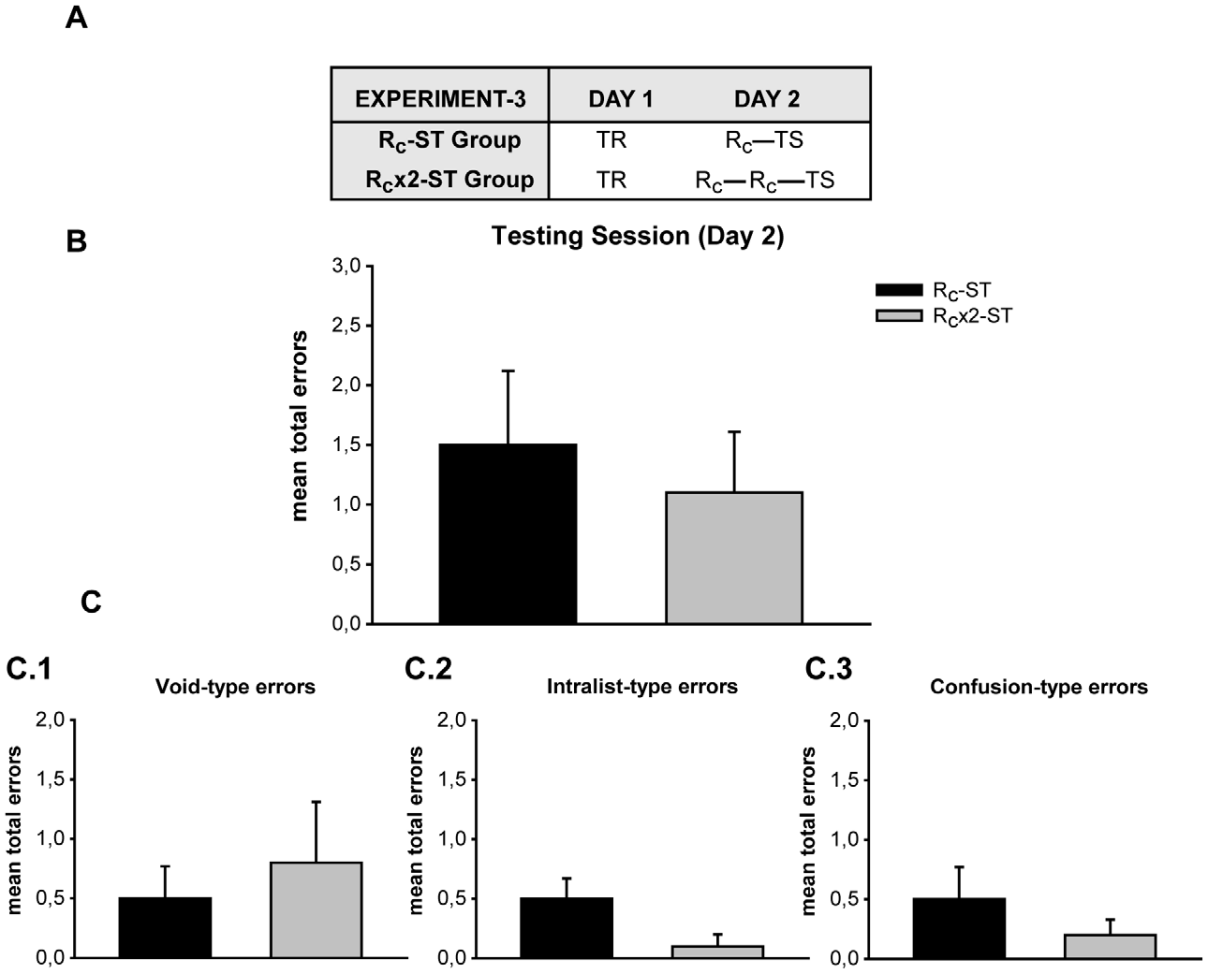
Experiment 3 (n = 10). Memory strengthening by repeated labilization processes is not expressed before reconsolidation takes place. **A**) **Experimental protocol.** A two-day experiment. On Day 1 subjects received the training (TR), on Day 2 they received the cue reminder (R_c_) before being tested (TS). Group R_c_-ST received a cue reminder, Group R_c_x2-ST received the cue reminder twice. **B**) **Testing session.** Mean number of total errors +/− SEM on Day 2. Black bar stands for Group R_c_-ST, grey bar for Group R_c_x2-ST **C**) **Error Type.** **C.1)** Mean number of Void-Type errors +/− SEM on Day 2. **C.2)** Intralist-type errors **C.3)** Confusion-type errors. Symbols as above.

Here again similar training performance for the R_c_ Group and the R_c-r_ x2 Group was revealed (Figure S1C, F(1,18) = 0,005 p = 0,943, interaction F(8,144) = 0,641 p = 0,743. *Inset*: F(1,18) = 0,240 p = 0,630). Subjects that received one cue-reminder or two cue-response reminders made a similar number of errors at the two testing trials on Day 3. Specifically, no significant differences were revealed at testing (Figure 2B F(1,18) = 0,462 p = 0,505). Moreover, in this case the comparison between the error types showed an equivalent number of errors for each type in both groups (Figure 3C; Void-Type: F(1,18) = 1,923 p = 0,182; Intralist-Type: F(1,18) = 0,000 p = 1,000; Confusion-Type: F(1,18) = 0,101 p = 0,754).

Therefore, successive retrievals on Day 2 did not improve the retention of the memory and this effect depends on the repetition of the presentation of reminders that induce the destabilization of the declarative memory.

### Memory strengthening by repeated labilization processes is not expressed before reconsolidation takes place

In order to assert that memory reconsolidation effects are at play, it needs to be shown that the post-reactivation manipulation is not effective shortly after the treatment, when the memory is unstable but intact [34]. Thus, to estimate the effect of two successive labilization processes on the strengthening of the target memory, the test session was done immediately after the treatment session. Hence, we carried out a two day experiment which involved two groups (Figure 3A). On Day 1, subjects learned a list of syllable-pairs (List). On Day 2, they received a treatment session. The cue-reminder short-term group was exposed to one cue-reminder (R_c_-ST) and the two cue-reminders group short-term (R_c_x2-ST) received the reminders twice separated by a 5-minute interval. Both groups were tested immediately after the presentation of the reminders.

#### Two cue reminders do not enhance performance when it is evaluated immediately after their presentation

As in previous experiments, an ANOVA of repeated measures revealed no significant differences between groups at training (Figure S1D. F(1,18) = 0,010 p = 0,923), and in comparison, no group trial interaction was found (F(8,144) = 0,876 p = 0,539). Moreover, no significant difference between groups for the percentage of correct responses for the last four training trials was disclosed (F(1,18) = 0,067 p = 0,798 *inset*).

Subjects that received one cue-reminder or two cue-reminders made a similar number of errors at testing when the two testing trials were given immediately after successive reactivations. In particular, no significant differences were disclosed at testing (Figure 3B F(1,18) = 0,207 p = 0,654). Besides, the comparison between the error types showed the same number of errors for each type in both groups (Figure 3C; Void-Type: F(1,18) = 0,269 p = 0,610; Intralist-Type: F(1,18) = 4,235 p = 0,054; Confusion-Type: F(1,18) = 1,000 p = 0,331).

Therefore, as was demonstrated in other paradigms and models when the effect of the treatment to impair or improve the reconsolidation process is evaluated immediately after its presentation, the memory is not affected. Here, the treatment provided was two successive cue-reminders which in turn triggered two consecutive labilization processes on Day 2. This experimental manipulation did not improve the performance when tested immediately after its presentation. In agreement with the general observation, the effect of the treatment depends on the completion of the re-stabilization process [34].

### The strengthening effect of repeated reactivations only appears when the second labilization occurs in the time window of the first

Up to this point, the improvement in the memory retention as a consequence of a double reactivation occurred when the cue-reminders were given successively in the same treatment session. In real life, outside the laboratory, it should be expected that the reactivations occur from time to time, and not necessarily one immediately after the other. So the remaining two experiments explored the effect of successive reactivations induced by the presentation of the cue-reminders separated by different time-intervals. To design these experiments we used two properties previously delineated for this paradigm. First, as we have shown in a previous study [29], the interfering task given 5 min or 6 h after presenting the reminder impairs the target memory acquired 24 hours before. On the contrary, the same training given 10 h after the reminder leaves the first memory intact, suggesting a reconsolidation period of at least 6 h. Second, another prior outcome used here is that the labilization-reconsolidation is no longer observed if the cue-syllable is omitted and only the context cues are presented (context-reminder, R_ctx_), nor if the possibility of answering with the corresponding response-syllable (cue-response reminder, R_c-r_) is added. In both cases the memory remains stable. So, in line with these precedents we chose for the first experiment a 24-hour interval, when the memory has recovered stability after the first labilization- reconsolidation process, and in the second experiment a 2-hour interval in which the second reminder was included in the depicted time window of the first. Moreover, in the last experiment we combined the reminder which induced labilization with a second, given two hours later which triggered retrieval only. Thus, we performed a four day experiment and a three-day one.

**Figure 4:**
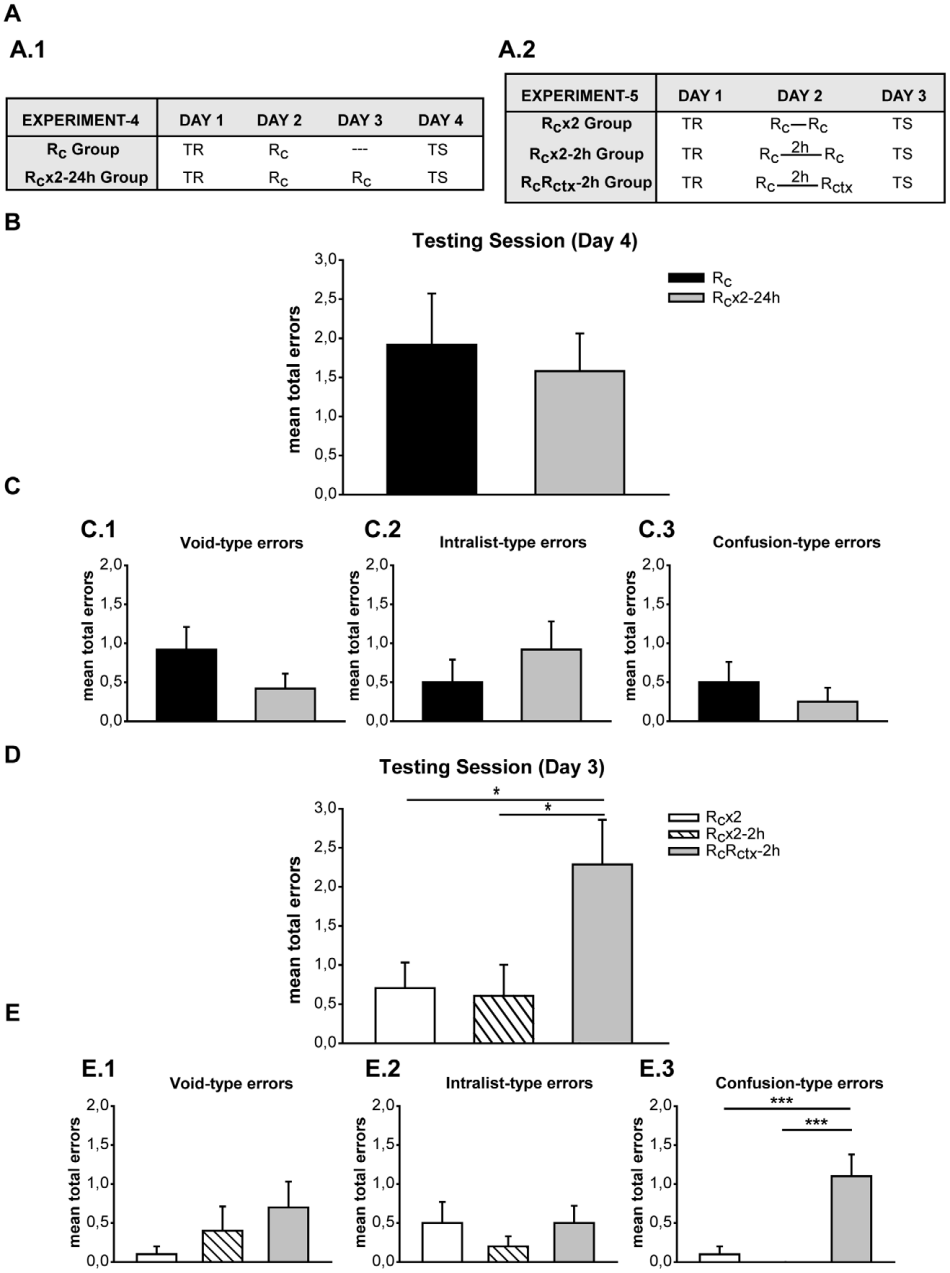
Experiment 4 (n = 12) and 5 (n = 10). Strengthening effect of repeated reactivations only appears when the second labilization occurs in the time window during the first. **A**) **Experimental protocols.** **A.1)** A four-day experiment. On Day 1 subjects received the training session (TR), on Day 2 they received the cue reminder (R_c_), on Day 3 only one Group received the cue reminder, and subjects were tested on Day 4 (TS). Group R_c_ received a cue reminder on Day 2, but received no treatment on Day 3. Group R_c_x2-24h received the cue reminder on Day 2 and 3. **A.2)** A three-day experiment. Symbols as in experiment 1, R_ctx_ stands for the context reminder. Groups differ in the number of reminders that they received on Day 2. Group R_c_ received a cue reminder, Group R_c_x2 received two cue reminders separated by 2 hours, and Group R_c_R_ctx_-2h received a cue reminder and a context separated by 2 hours. **B**) **Experiment 4, testing session.** Mean number of total errors +/− SEM on Day 4. Black bar stands for Group R_c_, grey bar for Group R_c_x2-24h. **C**) **Experiment 4, error type.** **C.1)** Mean number of Void-Type errors +/− SEM on Day 4. **C.2)** Intralist-type errors **C.3)** Confusion-type errors. Symbols as above. **D**) **Experiment 5, testing session.** Mean number of total errors +/− SEM on Day 3. *, p<0,05. White bar stands for Group R_c_x2, striped bar for Group R_c_x2-2h and grey bar for the Group R_c_R_ctx_-2h. **E**) **Experiment 5, error type.** **E.1)** Mean number of Void-Type errors +/− SEM on Day 3. **E.2)** Intralist-type errors **E.3)** Confusion-type errors. Symbols as above. ***, p<0,001.

In the first experiment, the R_c_ group learned the list of syllable-pairs (List) on Day 1. On Day 2 subjetcs received a cue-reminder and were tested on Day 4. The R_c_-24h group received the treatment session separated by 24 h-interval (Figure 4A1) and were finally tested on Day 4.

For the second experiment on Day 1, subjects learned the list of syllable-pairs (List). On Day 2 they underwent the treatment session (Figure 4A2). The two cue-reminders group (R_c_x2) was exposed to two cue-reminders separated by a 5 minute-interval and the two cue-reminders two-hour group (R_c_x2-2h) received the cue-reminders twice separated by a 2 hour-interval and the cue-reminder plus context-reminder group (R_c_R_ctx_-2h) were exposed to a cue-reminder and 2 hours later to a context-reminder (they received only the context cues). Finally, all groups were evaluated on Day 3.

#### The effect of a second cue-reminder given at diverse intervals after the first reminder presentation on reconsolidation of List memory

In both experiments as in previous ones, the number of correct responses acquired by all groups at training was similar for each group as was shown by an ANOVA of repeated measures (Figure S1E, F(1,22) = 0,815 p = 0,376; Figure S1F F(2,27) = 0,612 p = 0,550), and no group trial interaction was found (F(8,176) = 1,611 p = 0,125; F(16,216) = 0,307 p = 0,996 respectively). Furthermore, no significant difference between groups for the percentage of correct responses for the last four training trials was found (F(1,22) = 1,158 p = 0,294, F(2,27) = 0,506 p = 0,608 *inset*s, respectively).

With regard to the reactivations decoupled 24 hours, subjects that received one cue-reminder or two cue- reminders separated by one day made a similar number of errors at testing on Day 4. Particularly, no significant differences were disclosed at testing (Figure 4B F(1,22) = 0,171 p = 0,683). In addition, the comparison between the error types exhibited the same number of errors for each type in both groups (Figure 4C; Void-Type: F(1,22) = 2,084 p = 0,163; Intralist-Type: F(1,22) = 0,821 p = 0,375; Confusion-Type: F(1,22) = 0,622 p = 0,438).

In the last experiment where cue-reminders were given one immediately after the other (R_c_x2) or with a 2 hour- interval (R_c_x2-2h) or a cue-reminder and 2 h later a context-reminder (R_c_R_ctx_-2h), the performance on Day 3 revealed that groups with two cue-reminders presented successively or separated by two hours on Day2 showed a better performance than the group treated with a cue-reminder and two hours later a context-reminder (Figure 4D). In particular, the R_c_x2 and R_c_x2-2h groups made fewer errors than the R_c_R_ctx_-2h Group at the two testing trials (One-Way ANOVA F(2,27) = 4,500 p = 0,021; Post-hoc LSD Comparison p = 0,018, p = 0,013 respectively). Here again, It is worth stressing the difference exposed for the type of errors made by each group. Void type errors (blank responses, Figure 4E1 F(2,27) = 1,253 p = 0,302) were comparable for the three groups like the intralist type (write down a response syllable for another cue one. Figure 4E2 F(2,27) = 0,643 p = 0,534). However, R_c_x2 and R_c_x2- 2h groups made fewer confusion type errors (write down a nonexistent response syllable) than the R_c_R_ctx_-2h group. Indeed, significant differences were revealed between groups at the two test trials (Figure 4E3 One-Way ANOVA F(2,27) = 12,808 p = 0,0001; Post-hoc LSD comparison p = 0,0003, p = 0,0001 respectively). Taken together these experiments support the view that 2 labilization-reconsolidation processes strengthen the target memory when the second occurs in the time window of the first (R_c_x2 or R_c_x2-2h). On the contrary, when the first reconsolidation was finished and a second cue-reminder was presented (R_c_x2-24h) or when the second reminder provoked retrieval instead of reactivation (R_c_R_ctx_-2h), the memory maintained its original strength. In both cases, these groups showed a performance comparable with that exhibited by the group which underwent one labilization-reconsolidation process.

## Discussion

The central conclusion of this paper is that when memory is labilized by the presentation of the proper reminder and the process is again triggered by the presentation of another cue-reminder in the time window of the first, subjects can improve their performance at testing. Thus, we revealed that at least two labilization-reconsolidation processes reinforce the reactivated human memory. This outcome is asserted by the fact that the use of two cue-response reminders, which includes one syllable-response in its structure and prevents labilization, hinders the strengthening of the original memory. Thus, this result supports the view that the mere retrieval (induced once [32]; or as we did here twice by the presentation of two cue-response reminders) does not affect the stability of the retrieved memory, which is invulnerable to different treatments. The improvement depends on the re-stabilization process, which only occurs when the parametrical conditions of reconsolidation are fulfilled [32], [33]. Here it is necessary to stress a central result demonstrated throughout this paper: that two simple recalls (R_c-r_X2), which imply the absence of labilization, hinder memory enhancement. Moreover, the same effect can be obtained with the combination of a labilization-recall treatment (R_c_R_ctx_-2h). As a result, this strengthening phenomenon cannot be explained by the effects of simple retrievals, two labilization processes being an indispensably condition for memory reinforcement. Finally, this enhancement effect is revealed only after reconsolidation has occurred. Since, the performance maintained similar levels of errors when the test occurred immediately after the two-reminder presentations.

As we have done in previous studies, we analyzed error types. Here, it could be expected that after improvement, when the reinforcement process has taken place, there would be either a corresponding reduction in all the number of error types or a reduction in the number of some error types. It is noteworthy that the groups which received at least two cue-reminders showed fewer confusion errors than the other groups. To analyze how the improvement in performance is expressed via the decrease in this type of error, an initial categorization of confusion errors showed that the majority of errors made by subjects included typing three wrong letters, one wrong letter in a group of three or three correct letters but in the wrong order. Thus, the memory strengthening allowed the subjects to remember the three letters and their order, showing in a more insightful manner the improvement in their performance. In other words, the improvement in correct responses was a consequence of a decrease in confusion errors, shown by an increase in the precision of the memory. It would also be expected that the strengthening of the original memory by repeated labilization-reconsolidation processes maintain the memory available for longer periods as was demonstrated in rats for an aversive memory [28]. Further experiments with longer intervals between successive reactivations and the testing session may reveal whether the strengthening not only modifies the precision but also the duration of the memory. To design this experiment, we would consider previous results showing that a 7-day training-to-testing interval diminished the performance only by passing of time [35]. Thus, in this protocol combining repeated reactivations on Day 2 and 7-day training-to-testing interval it could be possible to evaluate whether only repeated reactivations improve the weakened memory with the passage of time.

At this point, it is necessary to undertake a more detailed analysis of the common components between the traditional animal models used in the literature to study memory reconsolidation and our human paradigm to characterize the same process. Animal models used to study reconsolidation are associative learnings, mainly conditioned fear paradigms. Thus, the animals acquire a conditioned response which is elicited when the animals are confronted with the conditions stimulus, during the reactivation session or at testing [34], [19]. The more useful reactivation session implies the presentation of only CS, and the animals retrieve and perform the conditioned response (i.e. freezing) [34], [36]. The paradigm used in this study is quite different. It is also an associative one, since the volunteers associate the task of responding to the cue-syllable with the proper response-syllable in a specific special context. As was shown throughout our studies the impossibility of answering with the response syllable when the cue syllable has been presented in the proper context reactivated the consolidated memory [29], [32]. The common component in the reactivation used for animal models and our paradigm is the presence of mismatch, that is the incongruence between what is expected and what actually occurs [23], [32]. Generally, in animal models it is the absence of the unconditioned stimulus, and in our human-paradigm, the lack of the written-down response-syllable which is the respective mismatch component. Taken together, the studies with animal models reveal that such incongruence may be given by qualitative and quantitative differences [19], [34], [37] . Thus, the first manipulation implies that the reinforcement does not occur at all (reactivation in absence of the reinforcement), and for the second type of protocol implies the magnitude of the reinforcement is not fully predicted (reactivation plus a weak reinforcement) [38]. Thus, for animal and human models designated to study memory reconsolidation, the process is triggered by the violation of the expectation based upon prior learning. All in all, the mismatch is an essential component in order to initiate the labilization- re-stabilization process.

In reconsolidation protocols some cues of the training are presented in absence of the reinforcement. Therefore the possibility that the treatment used induced extinction instead of reconsolidation must be addressed. In other words, in associative memories the repeated presentations of the conditioned stimulus alone induces the formation of the extinction memory [39]. The formation of this type of memory implies a decline in the frequency or intensity of the conditioned response [9]. Consequently, considering that the reminder represents an extinction trial, it is possible that successive presentations of reminders or a long exposure to it triggers the formation of the extinction memory which coexists with the original one [20], [22], [40]. However, in this case even four reminders produce an improvement in the performance during testing instead of impairment in the recall of the learned syllables. To our knowledge there are no results at present where the repeated presentations of related stimuli induce an extinction declarative-memory. A clear example of this situation appears in the research carried out by Schiller et al. [41]. Using Pavlovian fear conditioning in humans as a model paradigm, they provided evidence that old fear memories can be updated with non- fearful information provided during the reconsolidation time window. As a consequence, fear responses – reflected by skin conductance – are no longer expressed. However, the declarative memory component – the recognition of the figure associated with the shock - remained intact. In line with these results, using the same paradigm but with a pharmacological approach; Kindt et al. [42] found that oral administration of propanolol before the reactivation of the fear memory erased the behavioral expression of the fear memory but not the declarative component. Thus, these studies provide evidence in humans that old fear memories can be updated with new information or a drug-treatment given during the reconsolidation time window, but without a profound change in their declarative content.

According to a widely held concept, the formation of long-term memories relies on a reactivation and redistribution of newly acquired memory representations from temporal storage to neuronal networks supporting long-term storage [43]. The standard model of long-term memory consolidation with regard to declarative memory events and facts [44]-[46] considered that during the offline periods the newly acquired memory traces are gradually redistributed to neocortical regions by strengthening cortico-cortical connections. Therefore, memories become increasingly independent from the integrity of hippocampal regions, a process called systemic consolidation. Human studies investigating reactivation patterns during sleep showed that brain regions activated during training of a non-declarative memory task are activated again during subsequent rapid-eye movement (REM) sleep [47]. On the other hand, learning of a declarative task reactivated the hippocampus during slow wave sleep (SWS; [48]). Using an olfactory stimulus to reactivate declarative memory during sleep, Rasch et al. [49] showed that participants who learned a visual-spatial learning task under the presence of an odour, and then were re-exposed to the odor during subsequent SWS distinctly improved later retrieval of the task. These results support the hypothesis that once an odor has become associated as the context of learned object locations, reapplication of the odor during subsequent SWS acts as a context cue that reactivates the new memories and thereby boosts their consolidation. In a recent study with the same paradigm [50], it was tested whether the principle of transient destabilization would apply equally to the memory reactivation during SWS or wakefulness. In this design, the odor cues associated with the acquisition of the task were presented 30 minutes after training either during SWS or wakefulness. In both situations the reactivation induced by the odor was followed by an interference task (similar to the original one) to probe memory stability. The results showed that reactivation during a wake state destabilized the memory, making it sensitive to interference. In contrast, reactivation during SWS immediately stabilized the memory, resulting in a memory resistant to interference. Neuronal signs of memory reactivation have been revealed also during the post-learning period of wakefulness [51]-[53].

Grounded on the above references, and considering that although transiently disturbing processes affect consolidation, destabilization after memory reactivation during wakefulness could provide the possibility of modifying the existing memory trace [54]. We propose to follow the same line of thinking used for consolidation and apply it to the reconsolidation process. Thus, present results represent the first demonstration that in a wake state and guided by the cue-reminder, repeated reactivations induce a strengthening of a previously consolidated memory, as the odor used as a context cue reactivates the spatial learning and improves memory consolidation during SWS. This reinforcement effect in a wake state seems to be impervious to the external inputs which imply a real danger of encountering conflicting information and, surprisingly, in this case these new data would not interfere with the initiated reconsolidation process.

At this point, it is clear that two different phases are included in so-called reconsolidation. The first step is reactivation which implies a destabilization of the consolidated memory, and then a process of restabilization which returns the memory to a stable state. Recent studies have begun to identify the molecular mechanisms underlying the restabilization of reactivated memory [55]–[59], and as a result the memory is insensitive to disruptive agents again.

A speculative analysis of our results creates the possibility that successive reactivations trigger repetitive labilization processes which in turn imply successive restabilization processes. As a result, the second restabilization is mounted on previous restabilization resulting in a repeated activation of molecular pathways, which lead to either a higher expression of the macromolecules necessary for or an increasing number of macromolecules available for the recovery of the stable state. To prove this hypothesis, a new design with an animal model has to be developed showing that a differential effect of repeated labilization provokes an increase in the number of modifications associated with the plastic state and correlated with a specific mechanism improved by repeated triggering of the process.

However, for the first phase, the mechanisms underlying the initial destabilization remain even more poorly understood [60]–[61]. Until now, the requirement of LVGCCs or CBI receptors and the degradation by polyubiquitination of postsynaptic proteins were the mechanisms associated with the destabilization process [25], [62], [63]. Up to the present, the relation between these molecular pathways and memory reinforcement has not clearly emerged, leaving open the possibility of a mechanistic relation between the improvement and labilization phase.

As we pointed out before, the Cognitive tradition considers memory as being a permanently reconstructive dynamic process. Reconsolidation provides a plausible neurobiological mechanism for explaining some of the dynamic properties of memory [64]. Thus, cognitive psychology research has demonstrated the malleability of human memory, showing that memory can be changed by use, either in strength or in contents (i.e. flashbulbs memories, [3]; misleading post event information [4]). Both types of modifications, which summarize the two putative functions for the reconsolidation process, can be explained by a post-reactivation plasticity and the subsequent stabilization process [65]. Consistently, and going back to the initial goal of this work recapitulated in the following question, what is the function of memory reconsolidation? We have demonstrated in this research paper and in a previous one that reconsolidation can be operative for the two non-mutually exclusive hypotheses [15]. First, that destabilization of the original memory after labilization allows the integration of new information in the background of the original memory [33]. Secondly, the results obtained here reveal that the labilization-reconsolidation process strengthens the original memory by means of successive labilization reconsolidation processes rather than by adding training when the memory is unstable again [25]. On the other hand, Inda et. al. [28] revealed the strengthening function using successive retrievals of young memories. A most important difference with our protocol is that they reactivated the memory three times with and inter-reactivation interval of two days, so in this case it not seems necessary to include the second or third reactivation in the time window of the first one. In our study, successive reactivations with a 24 h-interval do not strengthen the memory. The cause of such disparity could arise from different species and paradigms.

In the framework of the strengthening function, this study has delineated essential concepts for the reconsolidation process emphasizing its biological role. Thus, simple memory reactivations strengthen the original memory by the repetition of the reminder presentations, representing a more adjusted way to show that reactivations (similar to those described during sleep) could modify a previous consolidated memory. Moreover, this strengthening induced by repeated labialization shows central parametrical constrains given that the second labialization must have occurred in the time window of the first triggered process. As a consequence, the improvement does not occur every time the reactivation is repeated and some specific conditions need to be included for this to occur.

In this model of declarative memory, we have demonstrated that the presentation of a proper reminder could guide the same memory to strengthening with the same content, or to updating the information included in it. The fate of the memory depends on how and where the reactivation took place. If there is relevant information to be included accompanied by a specific instruction to include it, the memory goes through the process adding the new information (memory updating). On the other hand, if the scenario implies repeated labilization without the presence of new information, the original memory is strengthened (memory strengthening). In this framework, both functions are likely to be triggered if two cue-reminders, the list with the new information and the instruction to include them are given [33]. As a consequence of the combination of both functions not only is the original memory improved, but also the new information integrated into it is strengthened.

All in all, both functions play a crucial role in this process, that is, reconsolidation is not merely an automatic re-stabilization triggered after retrieval; it is a truly special process which represents an opportunity for adaptive modifications of stored information.

## Materials and Methods

### Subjects

One-hundred and thirty-three undergraduate and graduate students from Buenos Aires University volunteered for the study. Before their participation in the experiment, subjects provided written informed consent that had been approved by the Comité de Ética de la Sociedad Argentina de Investigación Clínica Review Board. Their ages ranged from 20 to 35, with a mean of 25. Each participant was randomly assigned to one of twelve groups.

### Procedure

Experiments took place in a dark room and were conducted using a personal computer. Each subject was provided with earphones and seated facing a monitor placed in front of a large screen on the back wall.

Basically, subjects had to learn a List of five pairs of nonsense-syllables presented on the monitor screen. In the first trial the List was shown and in the successive trials the five cue-syllables were presented and subjects had to write down the corresponding response-syllable. The List was associated with a specific context (light projected on a large screen, an image on the monitor screen; and a sound coming through the earphones).

**Figure 5:**
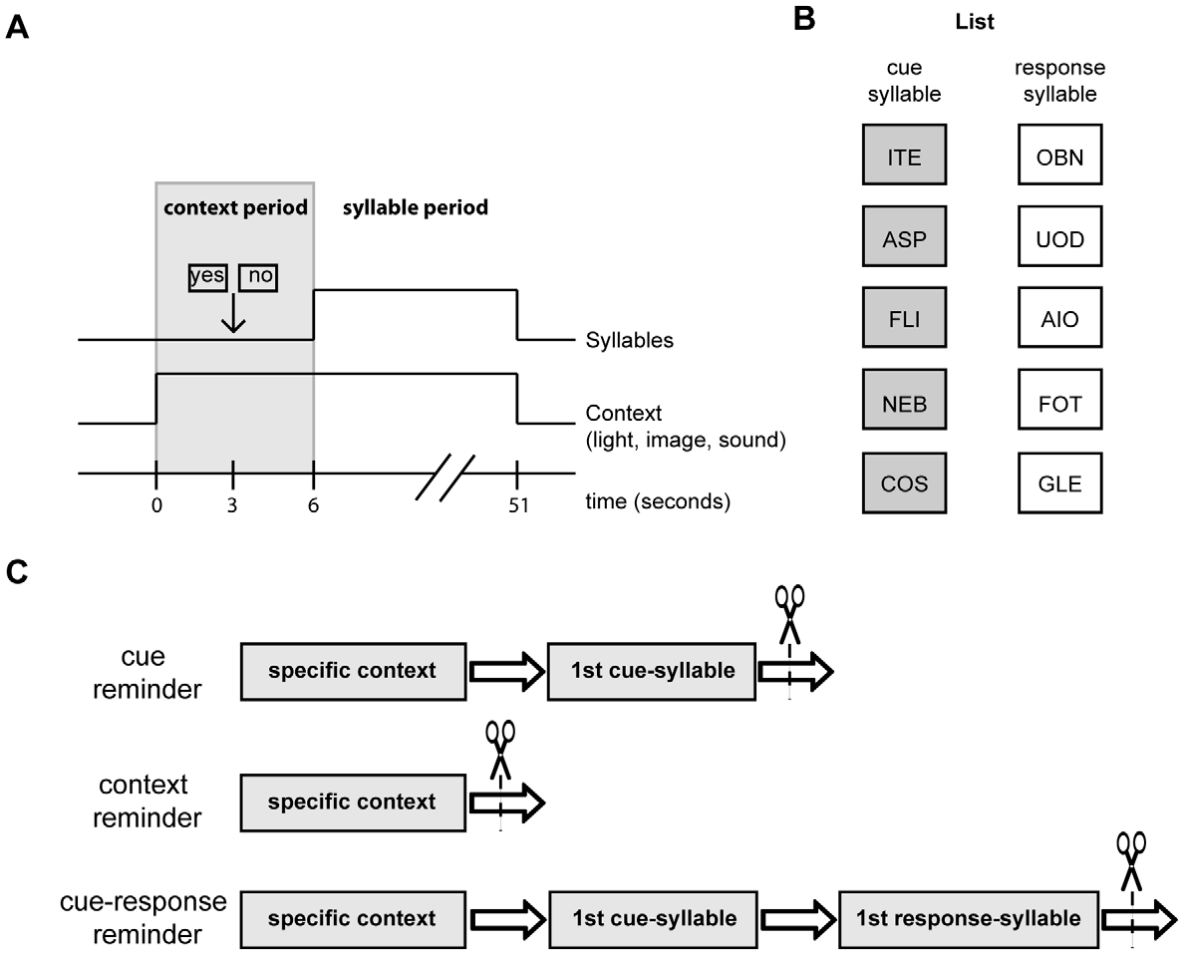
Experimental Protocol. **A**) **Actual trial.** It was formed by the context period: specific combination of a light (color illumination of the room), image (a picture on the monitor) and sound (music melody from earphones); and by a syllable period: six seconds after the stimuli presentation, five pairs of cue-response syllables were presented successively and in random order. **B**) **Paired-associated memory.** The List presented in the training and testing sessions. **C**) **Types of reminders.** ***(Top diagram)*** **The cue reminder (R_c_)** included the specific context, subjects had to press the expectancy keys (YES-NO), then one cue-syllable was presented after which the trial was abruptly interrupted, thus not allowing the subject to answer with the respective response-syllable**.** ***(Middle diagram)*** **The context reminder (R_ctx_)** consisted of the presentation of specific context, subjects had to press the expectancy keys (YES-NO) and the trial was abruptly interrupted before any syllable presentation. ***(Bottom diagram)*** **The cue-response reminder (R_c-r_)** included the specific context, subjects had to press the expectancy keys (YES-NO), then one cue-syllable was presented and subjects were allowed to write down the first response-syllable and after that the trial was interrupted. Scissors stand for the full-stop of each type of reminder.

There were two types of trials, actual trials (specific context + List) and fake trials (contexts that were never followed by the List presentation). Each trial began with the 6-second presentation of the context period (Figure 5A) but only actual trials were followed by the syllable presentation and the specific context, which persisted throughout (Figure 5A).

#### The training session

Each trial was composed of the **context period** with diverse stimuli options: the light could be blue or green; the image, three different pictures of cascades; the sound, three different tango melodies. Only one combination of these options (the specific context) was followed by the syllables presentation of List (syllable period). The trial which includes the specific context followed by the syllables presentation is termed the actual trial while the others with only context (i.e., without syllables presentation) are called the fake trials.

The **syllable period** started with the presentation of a cue-syllable on the left-hand side of the monitor screen and an empty response-box on the right. Each cue-syllable was taken at random from a list of five pairs. Subjects were given 5 s to write the corresponding response-syllable. Once that period was finished three situations were possible: first, if no syllable was written, the correct one was shown for 4 s; second, if an incorrect syllable was written, it was replaced by the correct one and it was shown for 4 s; and third, if the correct response was given, it stayed for 4 s longer. Immediately after that, another cue-syllable was shown and the process was repeated until the list was over. Altogether an actual trial lasted 51 s (6 s for context period and 45 s for syllable presentation). Throughout this paper, every time a subject faced a cue-syllable and wrote down an erroneous response or no response an error was computed.

The training consisted of the presentation of 10 actual trials mixed with 22 fake trials (total: 32 trials), separated by a 4-s intertrial interval. In the first training trial the List was shown, and in the successive actual trials subjects were required to write down the corresponding response-syllable for each cue-syllable presented. The List was composed of five pairs of nonsense cue-response-syllables in rioplatense Spanish: **ITE**-OBN, **ASP**-UOD, **FLI**-AIO, **NEB**-FOT, **COS**-GLE (bold type: cue-syllable; regular type: response-syllable) (Figure 5B).

Fake trials were presented in order to enhance the level of attention [32] and subjects were instructed to press the YES or NO button (the expectancy keys) on the keyboard 3 sec after the light–image–sound sequence had started (YES if they considered that it was the context associated to the List, NO in the opposite case). Therefore, this design allowed subjects to predict the presentation of the pair-associated task every time the specific context was completed.

Subjects that failed to obtain 60% correct syllable-responses during the block of the last four actual trials were excluded. The training session lasted 15 min.

#### Testing session

The testing session consisted of 2 actual trials mixed with 5 fake trials (total: 7 trials each). The testing session lasted 2,5 min.

An error was computed every time a subject faced a cue-syllable and wrote down an erroneous response or no response.

During testing we were allowed to record what subjects write down. Thus, to perform a more deeply analysis the errors executed at testing were classified in three categories: Void-Type error, when no response was written down; Intralist-Type error, when the response-syllable was not the right one but it belonged to the List; Confusion-Type error, when the response-syllable was not included in the List.

### Types of reminders

#### Cue reminder (R_c_)

This trial included the specific context, subjects had to press the YES or NO button (the expectancy key) and immediately after the context period, as expected, a cue-syllable appeared on the left-hand side of the monitor screen and the response-box on the right. However, 2 s later a notice displayed on the monitor announced that the session had to be suspended, thus not allowing the subject to write down the response-syllable (Figure 5C, Top diagram).

#### Cue-response reminder (R_c-r_)

This trial included the specific context, subjects had to press the YES or NO button (the expectancy key) and immediately after the context period a cue-syllable appeared and subjects were allowed to answer with the respective response-syllable. After that, a notice displayed on the monitor announced that the session had to be suspended (Figure 5C, Middle diagram). It was demonstrated that this type of reminder does not trigger memory labilization-reconsolidation [32], [33].

#### Context reminder (R_ctx_)

This trial included the specific context, subjects had to press the YES or NO button (the expectancy key) and immediately after the context period, before any syllable presentation, a notice displayed on the monitor announced that the session had to be suspended (Figure 5C, Bottom diagram). It was demonstrated that this type of reminder does not trigger memory labilization-reconsolidation [32].

### Demo

Before the training session, participants were confronted with a demo program to receive all the instructions and to understand the goal of the task. The program consisted of 4 trials, similar in structure to those of the training session, but with another context and two different pairs of nonsense-syllables.

### Experimental Groups

#### Experiment 1A (n = 13)

Group R_c_: Subjects received the training session on Day 1, the cue reminder on Day 2 and were tested on Day 3. Group R_c_x2: The protocol was the same as Group R_c_ but they received the cue-reminder two times separated by a 5-minute interval on Day 2. Group R_c_x4: Like Group R_c_ but subjects received the cue-reminder four times, each one separated by a 5-minute interval on Day 2.

#### Experiment 1B (n = 13)

Group R_c_: Subjects received the training session on Day 1, the cue reminder on Day 2 and were tested on Day 3. Group no-R: Subjects received the training session on Day 1 and were tested on Day 3.

#### Experiment 2 (n = 10)

Group R_c_: As in experiment 1. Group R_c-r_x2: Subjects received the training session on Day 1, two cue-response reminders separated by a 5 minute-interval on Day 2 and were tested don Day 3.

#### Experiment 3 (n = 10)

Group R_c_-ST: Subjects received the training session on Day 1, and on Day 2 they received a cue reminder and after a 5-minute interval were tested. Group R_c_x2-ST: Like Group R_c_-ST but subjects received the cue reminder two times separated by a 5-minute interval.

#### Experiment 4 (n = 12)

Group R_c_: Subjects received the training session on Day 1, the cue reminder on Day 2, and were tested on Day 4. Group R_c_x2-24h: Like Group R_c_-24h but subjects received one cue reminder on Day 2 and a second cue reminder on Day 3, to finally be tested on Day 4.

#### Experiment 5 (n = 10)

Group R_c_x2: As in experiment 1. Group R_c_x2-2h: The protocol was the same as Group R_c_x2 but subjects received the cue reminders separated by a 2-hour interval on Day 2. Group R_c_R_ctx_-2h: The protocol was the same as Group R_c_x2 but subjects received the cue reminder and context reminder separated by a 2-hour interval on Day 2.

### Statistics

#### Training Session

Mean number of errors per training-trial was reported and training curves were analyzed with repeated measures ANOVA.

#### Testing Session

Results were reported as mean number of total errors (block of first and second trial). Data from each experiment were first analyzed with one-way analysis of variance (ANOVA). It was followed by Post-hoc comparisons (FISHER, α = 0.05).

#### Types of errors

(Void, Intralist and Confusion-Types) were reported as mean number of errors (block of first and second trial) and were analyzed with one-way analysis of variance (ANOVA). It was followed by Post-hoc comparisons (FISHER, α = 0.05).

## Supporting Information

Figure S1 **Learning curves.** Mean number of errors +/−SEM per trial on Day 1. On the first trial the List is presented for the first time. A) Experiment 1A. Black dots stand for the Group R_c_, white triangles stand for the Group R_c_x2, white square for the Group R_c_x4. *Inset.* Mean number of total errors in the four last actual trials. Black bar stands for Group R_c_, white bar for Group R_c_x2 and stripe bar for the Group R_c_x4. B) Experiment 1B. Black dots stand for the Group R_c_, grey triangles stand for the Group no-R, Inset. Mean number of total errors in the four last actual trials. Black bar stands for Group R_c_, grey bar for Group no-R. C) Experiment 2. Black dots stand for the Group R_c_, grey triangles stand for the Group R_c-r_x2, Inset. Mean number of total errors in the four last actual trials. Black bar stands for Group R_c_, grey bar for Group R_c-r_x2. D) Experiment 3. Black dots stand for the Group R_c_-ST, grey squares stand for the Group R_c_x2-ST, Inset. Mean number of total errors in the four last actual trials. Black bar stands for Group R_c_-ST, grey bar for Group R_c_x2-ST. E) Experiment 4. Black dots stand for the Group R_c_, grey squares stand for the Group R_c_x2-24h, Inset. Mean number of total errors in the four last actual trials. Black bar stands for Group R_c_, grey bar for Group R_c_x2-24h. F) Experiment 5. White triangles stand for the Group R_c_x2, white dots stand for the Group R_c_x2-2h, grey squares for the Group R_c_R_ctx_-2h. Inset. Mean number of total errors in the four last actual trials. White bar stands for Group R_c_x2, striped bar for Group R_c_x2-2h and grey bar for the Group R_c_R_ctx_-2h.
(TIF)
