# Supplementary figures and images for: Repeated Labilization-Reconsolidation Processes Strengthen Declarative Memory in Humans
Source: PLoS One. 2011 Aug 5;6(8):e23305. doi: 10.1371/journal.pone.0023305 (PMC3151295; doi:10.1371/journal.pone.0023305)

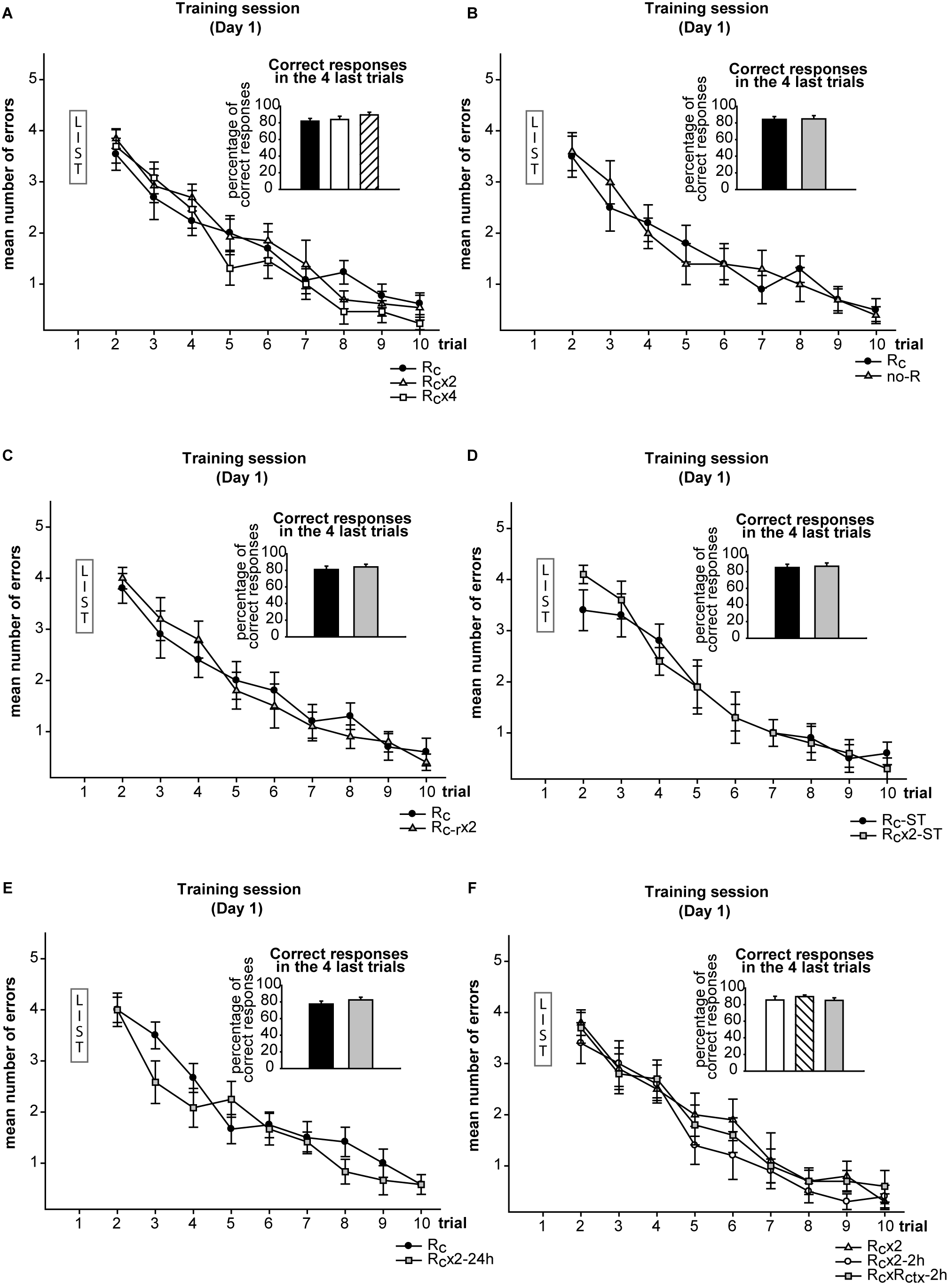

Supplement: Figure S1 — Learning curves. Mean number of errors +/−SEM per trial on Day 1. On the first trial the List is presented for the first time. A) Experiment 1A. Black dots stand for the Group Rc, white triangles stand for the Group Rcx2, white square for the Group Rcx4. Inset. Mean number of total errors in the four last actual trials. Black bar stands for Group Rc, white bar for Group Rcx2 and stripe bar for the Group Rcx4. B) Experiment 1B. Black dots stand for the Group Rc, grey triangles stand for the Group no-R, Inset. Mean number of total errors in the four last actual trials. Black bar stands for Group Rc, grey bar for Group no-R. C) Experiment 2. Black dots stand for the Group Rc, grey triangles stand for the Group Rc-rx2, Inset. Mean number of total errors in the four last actual trials. Black bar stands for Group Rc, grey bar for Group Rc-rx2. D) Experiment 3. Black dots stand for the Group Rc-ST, grey squares stand for the Group Rcx2-ST, Inset. Mean number of total errors in the four last actual trials. Black bar stands for Group Rc-ST, grey bar for Group Rcx2-ST. E) Experiment 4. Black dots stand for the Group Rc, grey squares stand for the Group Rcx2-24h, Inset. Mean number of total errors in the four last actual trials. Black bar stands for Group Rc, grey bar for Group Rcx2-24h. F) Experiment 5. White triangles stand for the Group Rcx2, white dots stand for the Group Rcx2-2h, grey squares for the Group RcRctx-2h. Inset. Mean number of total errors in the four last actual trials. White bar stands for Group Rcx2, striped bar for Group Rcx2-2h and grey bar for the Group RcRctx-2h. (TIF) [file pone.0023305.s001.tif]
